# Expanding the Scope of the Bumblebee Model BEE‐STEWARD: A Simple Foraging Module Facilitates the Parameterization

**DOI:** 10.1002/ece3.71468

**Published:** 2025-05-22

**Authors:** Max Luttermann, Reinhard Prestele, Volker Grimm, Jürgen Groeneveld

**Affiliations:** ^1^ Department of Ecological Modelling Helmholtz Centre for Environmental Research – UFZ Leipzig Germany; ^2^ Ammerländer Heerstraße 114‐118 Carl von Ossietzky Universität Oldenburg Oldenburg Germany; ^3^ Institute of Meteorology and Climate Research, Atmospheric Environmental Research (IMKIFU) Karlsruhe Institute of Technology Garmisch‐Partenkirchen Germany; ^4^ Plant Ecology and Nature Conservation University of Potsdam Potsdam Germany

## Abstract

The BEE‐STEWARD model simulates the population dynamics and behavior of bumblebees, including foraging, in remarkable detail, allowing the impact of various stressors on their populations to be assessed. To support the underlying detailed mechanistic descriptions, BEE‐STEWARD requires extensive parameterization, including corolla depth, which affects the handling time of foraging bees, for each flower species in the simulated landscape. However, this detailed approach limits the applicability of BEE‐STEWARD due to the lack of data for corolla depths, while also resulting in unrealistic foraging trip durations. Here we present a simplified foraging module that uses a constant handling time for foraging, thus eliminating the need to parameterize corolla depth. This simplification allows us both to apply the model to large scales and to assume handling times that reproduce observed foraging trip durations. Our new foraging module allows large‐scale population projections with BEE‐STEWARD. This increases its value in policy contexts and contributes to understanding and mitigating bumblebee declines.

## Graphical abstract

We present a simplified foraging model for the BEE‐STEWARD model that has been developed to simulate the population dynamics of bumblebees at a landscape scale. The new foraging model will facilitate the application and parameterisation of the model. In addition, the new foraging model results in handling times that are in line with empirical observations.

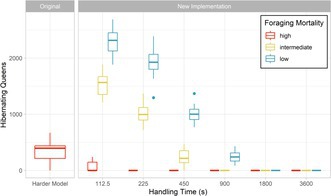

## 1 Introduction

Pollinators are confronted with numerous threats, many of which are associated with intensified land use, such as habitat loss and reduced landscape heterogeneity (Millard et al. 2021; Winfree et al. 2009). Studying these often interacting effects of stressors and projecting their impact on pollinators into real landscapes remains a difficult challenge (Goulson et al. 2002; Wood et al. 2015). Applying mechanistic models that are rich in structural realism and capable of incorporating real landscape data represents a promising approach to support empirical research (Becher et al. 2013). By incorporating landscape data and the full bumblebee life cycle, the BEE‐STEWARD model (Twiston‐Davies et al. 2021) became a suitable tool to explore and project individual and interacting stressors on bumblebee species in real landscapes (Baden‐Böhm et al. 2023; Knapp et al. 2019). Many of these stressors affect the foraging behavior of bumblebees, such as the spatio‐temporal distribution of foraging activity due to resource scarcities or decreased forage efficiencies as a consequence of pesticide exposure (Beyer et al. 2022; Feltham et al. 2014; Wang et al. 2024; Westphal et al. 2006). BEE‐STEWARD adapts the modeling approach of the honeybee model BEEHAVE (Becher et al. 2014; Twiston‐Davies et al. 2021) to simulate the population and colony dynamics of different bumblebee species in a landscape of approx. 4 × 4 km with a temporal resolution of 1 day. The landscape is characterized by the availability of nesting habitat and the spatiotemporal dynamics of the availability of floral resources. The model includes all major activities of bumblebees, such as nursing and foraging. By explicitly modeling the foraging process, BEE‐STEWARD allows us to explore the effects of various stressors on the foraging behavior of bumblebees.

However, applying BEE‐STEWARD to new landscapes can be challenging because it requires extensive parametrization. In addition to resource quantity and timing data, the model requires parameters for the corolla depth of each flower species. This corolla depth has been shown to impact the time needed for resource collection and is used by the model to estimate foraging durations (Balfour et al. 2013; Harder 1983). Foraging durations are a critical factor, determining not only exposure to foraging‐specific mortalities but also how many trips can be completed in a given period (Schmid‐Hempel and Heeb 1991; Stelzer et al. 2010). Thus, the foraging duration considerably affects the mortality risk and the amount of resources a colony can gather with potentially severe consequences for bumblebee population dynamics and persistence. However, the scarcity of data on the corolla depth parameter for many plant species, especially in the common floral resource databases (AgriLand (Baude et al. 2015), B‐GOOD (Filipiak et al. 2020), and FloRes (Baden‐Böhm et al. 2022)), makes its parameterization elusive. Additionally, the corolla depth underlies substantial variability across species, varieties, and individuals, which is furthermore unknown for most plant species (Atlagić et al. 2003; Cohen 2012; Ogishima et al. 2022). This variability and the limited knowledge about it prevent accurate and meaningful determination of the corolla depth parameter from literature data. Furthermore, the determination of foraging durations implemented in BEE‐STEWARD differs from the approach taken by the BEEHAVE model (Becher et al. 2014). The BEEHAVE model is a predecessor model of BEE‐STEWARD, using a similar modeling approach to simulate honeybee population dynamics. Both models can be used with the same landscape data. However, using a different approach to determine foraging durations prevents the reuse of landscape parameterizations of BEEHAVE projects. In addition, simulation experiments with BEE‐STEWARD revealed a substantial deviation in trip durations compared with empirical data.

We implemented a new approach to determine foraging durations to facilitate the application of BEE‐STEWARD, more specifically its parameterization. Instead of computing individual handling times for each flower, we use constant handling times for all foraging trips. This eliminates the need to parameterize the corolla depth and allows for easy integration of new landscapes. Our underlying goal was to broaden the scope of application for BEE‐STEWARD, allowing upscaling it to entire regions or even countries, as recently done for the BEEHAVE model (Becher et al. 2014; Groeneveld et al. 2024). Furthermore, we aimed to align the parameterization of BEE‐STEWARD with the BEEHAVE model. Thereby, we allow the reuse of landscape data and parameterization from previous BEEHAVE studies, reducing redundant parameterization effort and enabling researchers to focus on BEE‐STEWARD‐specific processes, such as nesting behavior. In addition, by directly controlling the handling times, our approach allows adjusting trip durations to more closely match empirical data. We conducted a set of simulation experiments to assess trip durations resulting from the original implementation and to test the new foraging module. We further assessed how the new foraging module affected trip durations and population sizes across various parameter combinations, highlighting changes in model behavior. Finally, we discuss how much our more generic and thus less realistic parameterization affects the uncertainty of the model predictions and how these uncertainties can be accounted for and reduced.

We would like to emphasize the exploratory nature of this study. The current limitations in our quantitative knowledge of bumblebee population dynamics make reliable calibration and validation almost impossible. Although BEE‐STEWARD's design is based on the widely used and tested BEEHAVE model, it is relatively new, and there are few BEE‐STEWARD studies available. We are not claiming that BEE‐STEWARD, either in its current state or with our new foraging module, is ready to be used for prediction. Rather, we are seeking to improve the usability of the model in order to progress to a state where it can be easily applied for thorough testing and validation, as has been and is being done with its predecessor model BEEHAVE.

## 2 Materials and Methods

The following sections describe how handling times are determined in the original BEE‐STEWARD model as published by Twiston‐Davies et al. (2021), the challenges resulting from this implementation, and the new foraging module that has been implemented to overcome these challenges. In addition, the experimental design of the simulations and the analysis conducted to assess and evaluate foraging trip durations resulting from the original and the new implementation are described.

### 2.1 The BEE‐STEWARD Model

BEE‐STEWARD is a mechanistic model designed to explore the colony and population dynamics of bumblebees in landscapes based on the spatiotemporal distribution of resources and nesting habitats (Becher et al. 2018; Twiston‐Davies et al. 2021). It is implemented in NetLogo (version 5.3.1, Wilensky 1999) and combines the two pre‐existing models Bumble‐BEEHAVE (Becher et al. 2018) and BEESCOUT (Becher et al. 2016). Thereby, it simplifies the previously disjointed workflows and facilitates the use by a wide range of stakeholders, such as researchers, policymakers, land management consultants, and practitioners. Bumble‐BEEHAVE and BEESCOUT are described in detail following the ODD‐Protocol in material linked to the original publications (Becher et al. 2018; Becher et al. 2016, see also beehave‐model.net). Here we only provide a summary description.

At the core of the BEE‐STEWARD model is the Bumble‐BEEHAVE model, which employs an agent‐based approach to simulate the behavior of individual (or cohorts of) bumblebees, which differ from each other regarding species, age, caste, size, and activity. The model encompasses all major activities of bumblebees, such as egg laying, nursing, and foraging, to describe their growth, behavior, and survival at all life stages. Thus, it provides structural realism for exploring the impact of various stressors, such as habitat loss and resource scarcity, aiming to disentangle their interacting effects at individual, colony, and population scales. Complementing this, the BEESCOUT model evaluates the spatial configuration of food resources and nesting habitats within the given map inputs. While Bumble‐BEEHAVE can simulate six different bumblebee species (individually or jointly), in BEE‐STEWARD, new species can be added via an input file (“BeespeciesFile”) without modifying the code, so the number and identity of species are not limited.

To initialize a simulation, a map section is imported, analyzed, and aggregated into food source patches. Afterward, an initial population of bumblebee queens is randomly placed within the landscape. Simulations start on January 1 with a daily time step. However, each individual has a daily time budget that can be distributed to different activities. After queens emerge from hibernation, they start searching for a suitable nesting habitat. In case a suitable nesting habitat is found, queens begin to form a colony by gathering the necessary amount of pollen and nectar to lay eggs. The activity of bumblebees belonging to a colony is determined by stimuli induced by colony needs, such as nectar requirements, which can lead to nectar foraging. Foraging bumblebees select food sources aiming to maximize their pollen and nectar intake rate. Food sources that have been successfully foraged will be memorized and taken into account in future foraging decisions. At the end of the colony development, new queens emerge, mate, and hibernate to repeat this life cycle the following year.

### 2.2 Theoretical Background of Handling Time in BEE‐STEWARD

Bumblebees forage for pollen and nectar. In BEE‐STEWARD, separate approaches are used to determine the durations of nectar and pollen foraging trips (Becher et al. 2018, ODD, pp. 97–99, the ODD is included in our Research Compendium see data statement for further details). In the following, we will focus on nectar foraging, as this process has given rise to several complications in the process of landscape parameterization (Section 2.2.2). The foraging procedure for pollen was left untouched, and pollen is, if not explicitly mentioned, excluded when talking about foraging in the following.

#### 2.2.1 Determination of Foraging Trip Durations in BEE‐STEWARD

Foraging trip durations (F) in the BEE‐STEWARD model are determined by the distance a bumblebee flies to a resource patch and back, plus the handling time ($T_{h}$) needed to gather a load of nectar from the patch (Equation 1). The flight time is the quotient of the distance (Δs) to (and from) the resource patch and the velocity of the bee (v).

(1) $$F=\frac{2\mathrm{\Delta s}}{v}+T_{h}$$

To determine the handling time of a foraging trip, first, the handling time per flower is calculated. This consists of the time to access the flower ($T_{a}$) and the time spent to travel between two flowers ($T_{t}$), both scaling with the filling level (γ) of the foraged resource patch, plus the time needed to ingest the available resources of a flower ($T_{i}$). Multiplied with the number of flowers needed to gather a load of nectar ($N_{\text{flowers}}$) this results in the total handling time for one load of nectar (Equation 2). To prevent unrealistic high handling times from almost emptied patches, handling times are bound to a maximum value ($T_{\max}$).

(2) $$T_{h}=\left\{\begin{array}{c} \left(\frac{T_{t}+T_{a}}{\gamma }+T_{i}\right)\times N_{\text{flowers}},T_{h}<T_{\max}\\ \;T_{\max\;},T_{h}\geq T_{\max} \end{array}\right.$$

The filling level γ represents the percentage of flowers of the according patch that are still filled with resources and the scaling of $T_{t}$ and $T_{a}$ thus represents the effect of resource depletion. While the time spent to travel between two flowers ($T_{t}$) is implemented as a constant and flower species‐specific parameter, the computation of the time needed to access ($T_{a}$) and the time to ingest the available resources of a flower ($T_{i}$) are determined according to models which Harder (1983) derived from lab experiments (Equation 3 and Equation 4). The access time ($T_{a}$) is determined by a linear function of the corolla depth (C) of the flower.

(3) $$T_{a}=0.3+0.04\;C$$

Besides the corolla depth (C), the function to determine the ingestion time ($T_{i}$) takes additional parameters into account, such as the nectar volume of the flower (V) and the weight (W) and glossa length (G) of the bumblebee (Equation 4, called Harder‐Equation in the following). The weight of the bumblebee is a state variable emerging during simulations, whereas the nectar volume, corolla depth and glossa length need to be parameterized by the user. While the nectar volume is used in multiple processes, the corolla depth and glossa length are exclusively used to obtain handling times.

(4) $$T_{i}=\frac{\log_{10}\left[V+1\right]}{\log_{10}\left[\frac{0.3\;W^{1/3}\;G}{{\left(1.41-C/G\right)}^{-0.4}-0.3T_{a}}+1\right]}$$

#### 2.2.2 Challenges of the BEE‐STEWARD Approach

Building on Harder (1983), the BEE‐STEWARD model mechanistically determines handling times, accounting for variations in weight and glossa length among bumblebee individuals and species, thus incorporating an intra‐ and interspecific competition mechanism. However, the extensive parameterization, specifically the data required to parameterize the corolla depth in order to derive handling times, limits the applicability of the model to landscapes other than those represented in Becher et al. (2018). Although a default parameterization is provided for the example maps included in the BEE‐STEWARD download (https://beehave‐model.net/download/), applying the model to new landscapes requires new parameterization.

Among the surveyed databases on floral resources, such as AgriLand (Baude et al. 2015) and B‐GOOD (Filipiak et al. 2020), none included information about the corolla depth. An exception was the FloRes database (Baden‐Böhm et al. 2022), but the available information was limited. Of the 168 plants cataloged, only 69 had entries for corolla depth, and many plant species common in agricultural landscapes were missing. Additionally, literature suggests that the corolla depth underlies a substantial variability among plant species of the same genus, or even varieties of the same species, for example, due to adaptations to local conditions such as pollinator communities (Cohen 2012; Ogishima et al. 2022). Consequently, data from individual studies are likely to provide only approximate estimates, as their results may be specific to the studied plant variety or geographical location. This issue is further complicated by the fact that some studies measure corolla length, while others measure corolla depth. Conversion between corolla length and depth requires a comprehensive understanding of floral morphology, and thus great caution is needed when selecting data for parameterization.

**FIGURE 1:**
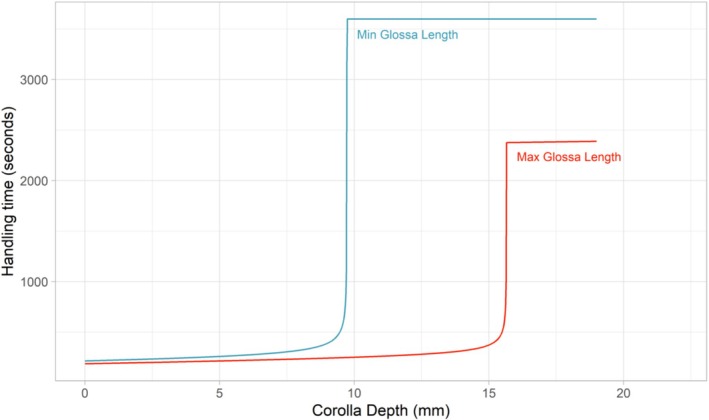
Bumblebee handling times for gathering a full load of nectar in BEE‐STEWARD. The depicted functions represent handling time ($T_{h}$) estimated for the minimum and maximum glossa length (G in Equation 4) of *Bombus terrestris* according to the default parameterization of BEE‐STEWARD (6.9 and 11.1 mm). Handling times were estimated using Equation (2) along the range of possible corolla depth values according to the default parameterization of BEE‐STEWARD (0 to 19 mm). The weight of the bee (W in Equation 4) was set to 0.195 g following Harder (1983) and the nectar volume (V in Equation 4) to the median value of all nectar‐providing flowers in the default parameterization. Note, that C and V are flower species specific and their combination is unique for each flower species. Furthermore, V, W, and G change during the simulation due to resource depletion and the growth of bees.

The problem of lacking knowledge about the corolla depth and the underlying variability is even further amplified by the nonlinear character of the Harder‐Equation (Figure 1). Close to the pole of the function, minor changes in the corolla depth–glossa length ratio result in substantial differences in the handling time. Applying the Harder‐Equation without the ability to determine the required parameters with sufficient accuracy is prone to induce more error rather than more realism.

Since BEE‐STEWARD originated from the BEEHAVE model (Becher et al. 2014) they share the same landscape parameterization, except for the corolla depth. Thus, landscape parameterizations for BEEHAVE cannot readily be reused for BEE‐STEWARD projects. An alternative approach to determine foraging durations without information on the corolla depth could benefit from the research including the parameterization effort that has been conducted for BEEHAVE, which was used in more than 25 published studies (Groeneveld et al. 2024).

### 2.3 The New Foraging Module for Handling Time in BEE‐STEWARD

To overcome the problems identified in the original implementation, we propose a new approach for determining handling times in the BEE‐STEWARD model. This new foraging module simplifies the determination of handling times, setting it constant, regardless of the different parameters used in the Harder‐Equation, such as the glossa length of the bumblebee or the corolla depth of the flower (Equation 4). Hence, these parameters are no longer needed, eliminating the necessity to parameterize them. However, it is important to note that, due to technical constraints, dummy values for glossa length and corolla depth are still in the parameter files, but these values are no longer consequential when using the new foraging module. Equally to the original implementation, the handling time (*T*_*h*_) is divided by the filling level (γ) and bound to a maximum value ($T_{\max}$ in Equation 5). Thereby, we account for resource depletion and the elimination of unrealistically high handling times resulting from almost emptied patches.

(5) $$T_{h}=\left\{\begin{array}{c} \frac{\text{FixedHandlingTime}\_s}{\gamma },\;\text{if}\frac{\text{FixedHandlingTim}e_{s}}{\gamma }<T_{\max}\\ \;T_{\max\;},\;\text{else} \end{array}\right.$$

The new foraging module was added to the original BEE‐STEWARD code within a conditional statement, using the Boolean parameter *FixedHandlingTime?*, which is included in both the General Parameter File and BEE‐STEWARD graphical user interface (GUI). When *FixedHandlingTime?* is set to TRUE, the handling time ($T_{h}$) for each foraging trip is assigned a constant value. This constant handling time is set by an additional parameter (*FixedHandlingTime_s*), also included in the General Parameter File and BEE‐STEWARD GUI. We tested different values for *FixedHandlingTime_s* (Section 2.4) to determine the most suitable values.

A comprehensive description of the new foraging module, including technical details, can be found in the updated ODD model description in the Supplementary Information.

### 2.4 Simulation Experiments and Analysis

We conducted simulation experiments to (1) compare foraging durations derived from the original model against empirical data and (2) evaluate the effects of the new foraging module on model behavior, specifically on foraging trip durations and population size under varied parameter settings, including the *FixedHandlingTime_s*. To run simulation experiments, we used the modified BEE‐STEWARD version (Section 2.3) and NetLogo version 6.2.2 (Wilensky 1999). If not stated otherwise, parameters were set according to the BEE‐STEWARD default values as provided with the BEE‐STEWARD download (https://beehave‐model.net/download/). We conducted simulation replicates for each combination of parameters, each running for 5 years (1825 timesteps). Guided by the experimental setup of Twiston‐Davies et al. (2021), simulations were carried out using the *Foodsources Files* of the Example Farm as map input with an initial bumblebee population of 100 queens of the species *Bombus terrestris* and no badgers. The Example Farm represented a realistic, resource‐poor landscape consisting of the habitat types semi‐improved pasture, maize, hedgerow, scrub, permanent pasture, and a small flower‐rich plot (Twiston‐Davies et al. 2021).

#### 2.4.1 Scenarios

We ran 19 scenarios to explore model behavior with the original implementation and the new foraging module. We named the scenarios following the syntax *mortalitymodel_handlingtime*, which reflects the scenario settings (Table 1). An exception is the scenario using the original model.

**TABLE 1:** Overview of scenario settings and names.

Scenario name	FixedHandlingTime?	FixedHandlingTime_s	ForagingMortalityModel
ORG	FALSE	None	High (default)
LOW_112	TRUE	112.5	Low
LOW_225	TRUE	225	Low
LOW_450	TRUE	450	Low
LOW_900	TRUE	900	Low
LOW_1800	TRUE	1800	Low
LOW_3600	TRUE	3600	Low
MED_112	TRUE	112.5	Intermediate
MED_225	TRUE	225	Intermediate
MED_450	TRUE	450	Intermediate
MED_900	TRUE	900	Intermediate
MED_1800	TRUE	1800	Intermediate
MED_3600	TRUE	3600	Intermediate
HIGH_112	TRUE	112.5	High
HIGH_225	TRUE	225	High
HIGH_450	TRUE	450	High
HIGH_900	TRUE	900	High
HIGH_1800	TRUE	1800	High
HIGH_3600	TRUE	3600	High

To test foraging durations resulting from the original implementation, the *FixedHandlingTime?* was set to FALSE and all other parameters were set to their default values. This scenario is called **ORG**.

To conduct simulations based on the new foraging module, *FixedHandlingTime?* was set to TRUE and additionally, *FixedHandlingTime_s* and *ForagingMortalityModel* varied. For the new *FixedHandlingTime_s* parameter, we used 112.5, 225, 450, 900, 1800, and 3600 seconds, to test model behavior for a broad range of values. A handling time of 3600 seconds corresponds to the maximum handling time ($T_{\max}$), meaning that resource depletion (γ) has no effect in these scenarios (Equation 5). The *ForagingMortalityModel* is a built‐in parameter determining mortality rates during foraging and was set to the three available values “high” (default), “intermediate,” and “low.” We varied the *ForagingMortalityModel*, anticipating a rapid extinction of the bumblebee population due to prolonged foraging durations, resulting in a very low number of foraging trips. *ForagingMortalityModel* = “high” corresponds to a mortality rate of $10^{-5}\;s^{-1}$ and was adapted from the BEEHAVE model and thus parameterized for honeybees (Visscher and Dukas 1997). The “intermediate” model is based on a study by Schmid‐Hempel and Heeb (1991), which measured the mortality rates for *Bombus lucorum* of $2.14\times 10^{-6}\;s^{-1}$ on a study site in Switzerland. When *ForagingMortalityMode*l = “low” foraging mortality rate is set to $2.75\times 10^{-7}\;s^{-1}$ based on a study by Stelzer et al. (2010), measured for multiple species including *Bombus terrestris* on multiple study sites including the UK and Germany. In the following, we will call these scenarios by the tested foraging mortality (HIGH, MED, LOW) followed by an underscore and the tested fixed handling time value (112, 225, 450, 900, 1800, and 3600 s).

The following metrics were recorded for all simulations in each timestep (day): the total number of queens, colonies, nectar, and pollen trips, the mean daily trip duration of all nectar forage trips, as well as the mean daily trip duration of all pollen forage trips. Recording the duration of each foraging trip was not feasible due to the extensive number of trips.

### 2.5 Analysis

Since we were interested in the mean trip duration of all foraging trips, we weighted the recorded daily means by the number of trips of the given day. Thus, to derive the measure “mean trip duration” we multiplied the daily mean trip durations by the corresponding number of trips per day, summed these products and divided the sum by the total number of trips across all days. The number of colonies in the last 3 years of each simulation was used to compare against the results of Twiston‐Davies et al. (2021). Further, we used the number of queens at the end of each simulation (December 31) as a proxy for the population size. Since empirical data for the comparison of such population measures are not available, we compared the results of the new foraging module with those of the original model. Comparison to literature was focused on the trip durations observed by Ings et al. (2006), who measured nectar foraging trip durations of *Bombus terrestris* in mid‐June on five different study sites in the UK. They selected study sites representing natural habitats of *Bombus terrestris* that included grassland, hedgerows, domestic gardens, and woodland and are therefore comparable to the map input used for simulations. Pollen foraging trips and orientation flights (trips shorter than 300 s) have been excluded in this study, thus only including nectar foraging trips in their analysis. Other studies also measured the foraging trip durations of bumblebees, but without distinguishing between nectar and pollen foraging trips (Table 2). The comparison therefore focuses on the study by Ings et al. (2006) and use it as a lower benchmark, since it presents the lowest mean value of all studies.

**TABLE 2:** Empirical data on foraging duration of bumblebees.

Source	Foraging duration in min	Resource	Info
Ings et al. (2006)	18–52	Nectar	Range of mean values for different study sites
Westphal et al. (2006)	66–82	Pollen and nectar	Mean values for landscapes with abundant and sparse resources
MacKenzie et al. (2021)	45 (±36)	Unclear	Mean (± standard deviation)
Spaethe and Weidenmüller (2002)	57–75	Pollen and nectar	Range of mean values for different colonies

## 3 Results

### 3.1 Trip Duration

**FIGURE 2:**
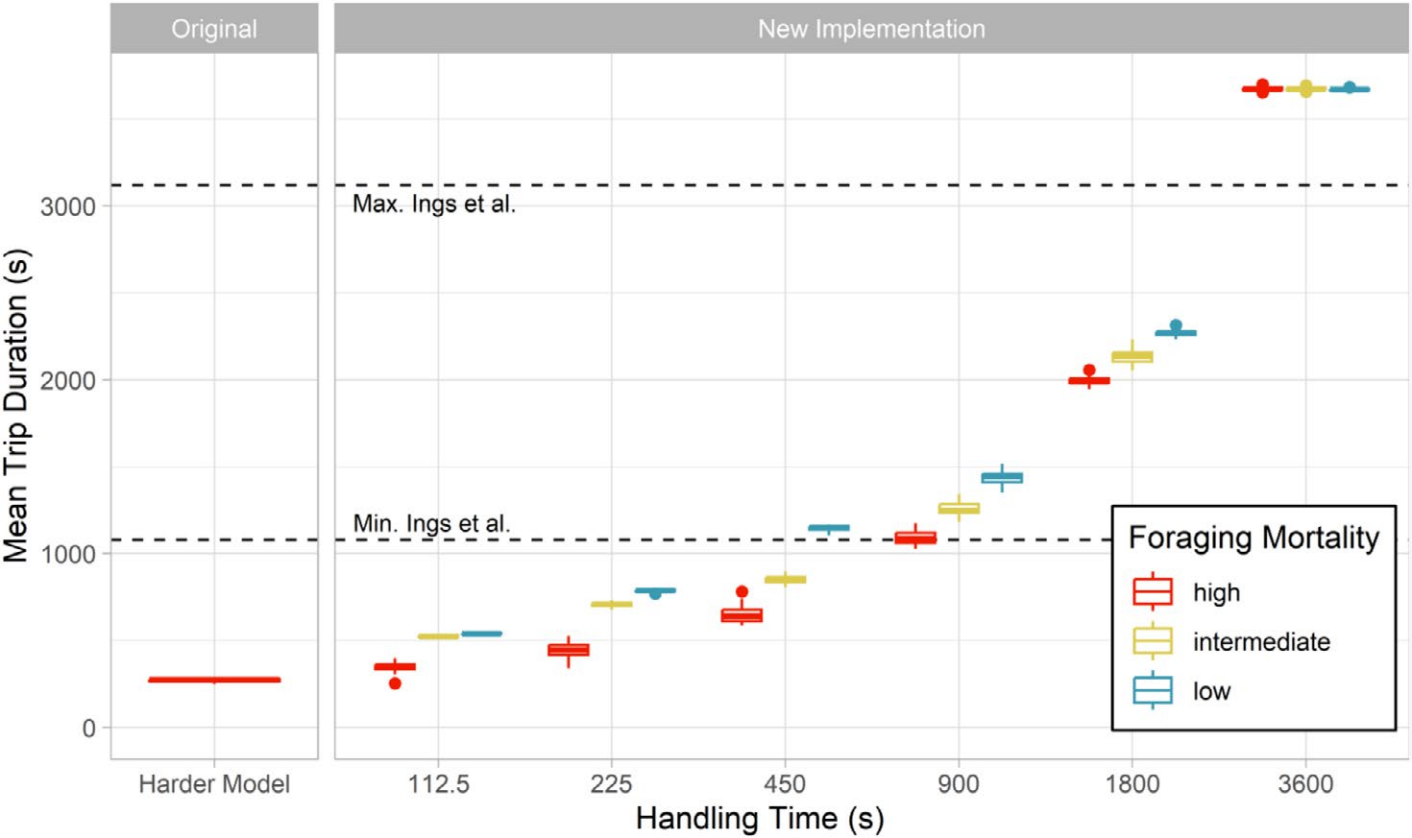
Mean trip durations (nectar foraging) for the original Harder model of BEE‐STEWARD and for different combinations of the new *FixedHandlingTime_s* parameter and foraging mortality. The original BEE‐STEWARD implementation was tested with the default parameterization (*ForagingMortalityModel* = “high”), while the new foraging module was tested across varying foraging mortalities (low, intermediate, and high), indicated by boxplot colors. The dashed lines represent the range of mean trip durations observed by Ings et al. (2006) (Table 2).

The mean trip duration of all nectar foraging trips in the original BEE‐STEWARD implementation was shorter than any parameter combination tested for the new foraging module (Figure 2). For the original implementation (ORG), we found a mean trip duration for nectar of 270.64 s, which was shorter than the mean trip duration of pollen trips (435.03 s).

With the new foraging module, trip durations of nectar foraging trips differed substantially from those of the original implementation as well as among the different scenarios (Figure 2). Trip durations ranged from 347.91 s (for the scenario HIGH_112) to 3672.13 s (for the scenario LOW_3600) and increased linearly with the fixed handling time. Furthermore, trip durations decreased substantially with increasing foraging mortalities for all tested fixed handling times, except where it was set to the maximum handling time (*FixedHandlingTime_s* = 3600). Pollen trip durations, which are not directly affected by changes in *FixedHandlingTime_s*, on the contrary, were only slightly negatively correlated with the fixed handling times ranging from 421.99 s (for scenario HIGH_112) to 365.55 s (for scenario LOW_3600).

### 3.2 Population Sizes

Simulations of the original implementation (ORG) yielded a mean of 6.39 colonies and 351.6 hibernating queens (SD ±203.46 queens).

**FIGURE 3:**
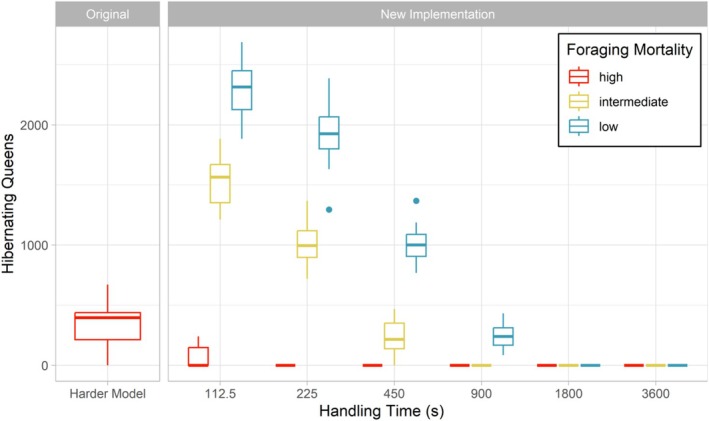
Number of hibernating queens for the Harder model of the original BEE‐STEWARD model, and for different combinations of *FixedHandlingTime_s* and *ForagingMortalityModel*. Boxplots represent the number of hibernating queens after 5 years (queens in the last timestep, 31 December, of the simulation) with 20 replicates for each scenario. The original BEE‐STEWARD implementation was tested with the default parameterization (*ForagingMortalityModel* = “high”), while the new foraging module was tested across varying foraging mortalities (low, intermediate, and high), indicated by boxplot colors.

Simulations using the new foraging module revealed that both the *FixedHandlingTime_s* and the *ForagingMortalityModel* substantially affected the population size (Figure 3). Population sizes ranged from 0 hibernating queens (for the scenario HIGH_3600) to 2294.4 hibernating queens (for the scenario LOW_112). The number of hibernating queens decreased with increasing fixed handling times. This correlation was found to be logarithmic. No population survived for fixed handling times of 1800 and 3600 seconds, regardless of the foraging mortality model. An increase in the foraging mortality resulted in a decrease in population sizes. Almost all populations went extinct using the new foraging module with high foraging mortality, regardless of the handling time.

Comparing the original and new implementation under the “high” *ForagingMortalityModel* setting, the ORG scenario showed larger population sizes than the scenarios using the new foraging module values (Figure 3). In contrast, the scenarios LOW_112, LOW_225, and LOW_450 yielded substantially larger populations compared with the ORG scenario. Notably, the scenarios MED_450 and LOW_900 yielded comparable population sizes to those of the ORG scenario.

## 4 Discussion

Our goal was to facilitate the parameterization of the BEE‐STEWARD model such that the integration of new landscape data becomes more feasible. Thereby, we aimed to ease the application of BEE‐STEWARD and allow upscaling to entire countries or regions, as recently done for the BEEHAVE model (Groeneveld et al. 2024). To achieve this, we implemented a new, simplified foraging module inspired by the approach from the BEEHAVE model.

### 4.1 The New Foraging Module Facilitates the Application of BEE‐STEWARD

Our new foraging module eliminates the need to parameterize corolla depth by using a fixed handling time for nectar foraging. Due to the limited availability of corolla depth data, its parameterization has been a major obstacle in applying the model to new landscape data. This was further complicated by the variability underlying corolla depth, which limits meaningful parameterizations without extensive fieldwork. Such fieldwork is not only impractical at large spatial scales but also favors site‐specific accuracy over broad applicability. However, broad applicability is an important feature for informing policy decisions (EFSA PPR Panel 2015). Eliminating the need to parameterize the corolla depth greatly facilitates using new landscape data, allowing us to upscale the model to large spatial scales. It also allows for the integration of existing land cover data and associated parameterizations from BEEHAVE projects (e.g., Agatz et al. 2023; Groeneveld et al. 2024). Thereby, the new foraging module enhances synergies between the two models, saving redundant work and allowing us to focus on the unique features of the BEE‐STEWARD model, such as nesting behavior. At the same time, the new foraging module maintains the generalizability of BEE‐STEWARD, allowing us to use it for different bumblebee species and any mapped landscape.

### 4.2 Foraging Durations of the Original Model Do Not Match Empirical Data

Furthermore, our simulation experiments showed that the foraging durations determined by the original BEE‐STEWARD are substantially lower than those observed by Ings et al. (2006), and consequently, lower than any other value found in the literature (Table 2). The discrepancy between the simulated foraging duration and the literature values may be caused by differences between the studied landscapes. For example, abundant resources have been shown to reduce foraging durations (Harder 1983; Westphal et al. 2006).

However, the landscapes used in our simulations were characterized as “resource‐poor” (Twiston‐Davies et al. 2021), whereas the landscape description provided by Ings et al. (2006) suggests abundant resources, which is contradictory to the explanation that the short foraging durations are related to the resource abundance. In general, a more thorough analysis of the landscape dependencies of foraging durations would improve the robustness of the obtained results. Ideally, this would be done by comparing the simulation results of the original model on a comprehensive set of maps with empirical data of the same geographic location. However, this would require extensive parameterization including the corolla depth, which was beyond the scope of this study. However, we found a mismatch between simulated foraging durations and empirical data for all example maps provided with the BEE‐STEWARD model download. Additionally, most of the foraging durations simulated with the original model were shorter than 300 s, which is commonly used as a threshold in the empirical literature, below which trips are considered orientation flights rather than foraging trips (Capaldi and Dyer 1999). Therefore, most simulated trips would have been excluded from empirical studies. Both the deviation from empirical data and the overall short foraging durations suggest a systematic underestimation of foraging durations in the original model.

Another explanation for the deviation of simulation results of the original model and empirical data could be an underestimation of the time spent traveling between two flowers ($T_{t}$ in Equation 2). This was implemented as a flower species‐specific parameter but is by default set equally for all flower species. This duration is expected to vary substantially depending on the density of each flower species and its characteristics (e.g., solitary flowers vs. inflorescence). The impact of this parameter and possible parameterizations to achieve a better match between simulated foraging duration and empirical data was not explored in this study. Investigating these aspects could be a valuable direction for future research.

Furthermore, our findings indicate that the duration of nectar foraging trips was shorter than that of pollen trips. This is contrary to the conclusions of previous studies on honeybees (Crenna et al. 2020, Table S4 and S5; Winston 1991, p. 173). If this also applies to bumblebees, it would provide additional support for the underestimation of nectar foraging durations by the original model.

In summary, while the time to gather resources is in general highly variable (Table 2 and Rodney and Purdy (2020) Table S2 in supplementary material 3), none of the points discussed fully explain the discrepancy between the simulated foraging duration of the original implementation and the empirical data.

### 4.3 Model Behavior With the New Foraging Module

With our new foraging module, users gain more control over the foraging duration. Our simulation results demonstrate the pronounced effect of the fixed handling time on the foraging durations. By adjusting the new *FixedHandlingTime_s* parameter, it was possible to match the foraging durations with empirical data. For example, scenarios with a *FixedHandlingTime_s* of 900 and 1800 s yielded foraging durations within the range of durations observed by Ings et al. (2006) (Figure 2). Furthermore, the scenarios with a *FixedHandlingTime_s* of 3600 s resulted in foraging durations that are in the range of the other empirical data (Table 2). The linear scaling of trip duration with increasing fixed handling time was expected given the additive approach (Equation 1). This linear scaling will cease once the fixed handling time exceeds the maximum handling time (*FixedHandlingTime_s* $\geq T_{\max}$). In light of the empirical foraging durations (Table 2), which in part exceed 3600 s, it may be necessary to reconsider the default value for $T_{\max}$.

In BEE‐STEWARD bumblebees optimize foraging with respect to energetic efficiency based on undertaken foraging trips (Becher et al. 2018, ODD, pp. 22, the ODD is included in our Research Compendium see data statement for further details). As shown, our new foraging module might alter foraging durations. Foraging efficiencies are further altered since handling times are no longer floral species‐specific.

Besides affecting the foraging duration, the fixed handling time also impacted the population size (Figure 3). Increasing handling times led to substantially lower population sizes. This was anticipated given that an increase in the duration of one foraging trip reduces the number of trips a forager can complete in a given period and consequently also the total amount of resources it can gather. Additionally, longer handling times also prolong the time foragers are exposed to foraging mortality. Notably, almost all populations went extinct for almost any tested fixed handling time value in combination with the default foraging mortality (“high”).

Likewise, the chosen foraging mortality substantially affected the population size (Figure 3). Interestingly, the foraging mortality also decreased the foraging duration (Figure 2). This decrease in trip duration with increasing foraging mortality is assumed to be related to the differences in the population sizes. Higher foraging mortalities result in lower population sizes, leading to less resource exploitation, meaning higher filling levels and thus less prolonging the handling time (Equation 5). This is supported by the results of the scenarios *_3600, in which trip durations remain unaffected by resource exploitation (Section 2.4), and no substantial difference in foraging duration was found for different foraging mortalities. In general, the observed pattern that resource gathering takes longer with less abundant resources is well in line with the literature (Harder 1983; Westphal et al. 2006) and justifies the scaling of the handling time with the filling level.

While pollen foraging durations were shorter than those for nectar for the original implementation, this relationship reversed with increasing fixed handling times. The fact that nectar foraging takes longer than pollen foraging is generally consistent with the literature, but the newly introduced difference between nectar and pollen foraging durations found in scenarios with a fixed handling time of 900 s and longer is likely too large (Rodney and Purdy (2020) Table S2 in supplementary material 3). An in‐depth analysis of pollen handling times, similar to this study, would be advisable and may necessitate a revision of this process.

Our results demonstrate changes in model behavior over a range of parameter settings. However, which parameter settings are most realistic remain an objective for future research. Currently, validation of resulting population sizes is limited by the lack of empirical data and are restricted to comparisons with the original BEE‐STEWARD model. Given that the foraging mortality model “high” is based on a study of honeybees, it is likely to overestimate the foraging mortality for bumblebees. The use of “medium” or “low” foraging mortality is therefore not only necessary to maintain populations with the new foraging module but also ecologically more realistic. However, even with the foraging mortality “low,” populations could not survive with fixed handling times of 1800 and 3600 s, while these scenarios still resulted in realistic trip duration values consistent with empirical data (Table 2). Using the new foraging module, scenarios LOW_900 and MED_450 yielded population sizes similar to the original model while showing foraging durations within the range of Ings et al. (2006).

We cannot recommend using a specific value for *FixedHandlingTime_s*, as neither existing direct measurements of this parameter nor existing population‐level data for calibration are sufficient for narrowing down this parameter to a single value. Running the model for settings between LOW_900 and MED_450 is recommended to emphasize the uncertainty in model predictions due to insufficient data. Our new foraging module nevertheless is a major simplification, as the original module would have required, for each new region, to determine 10–50 or more corolla depth parameters for which no or potentially highly site‐specific data exist.

### 4.4 Limitations

Our new foraging module represents a strong simplification, where we have simplified the complex interplay to one generic handling time for all foraging trips. Given the complex variation realized in nature, one fixed handling time value cannot be representative. However, this simplification makes the uncertainty underlying this process more explicit. Besides avoiding the necessity to pin down potentially highly variable parameters into one value, it also avoids the misconception that foraging durations can be precisely estimated once these parameters are known. Instead, our new foraging module allows one to choose handling times that are consistent with the available data, thus showing more realism besides being much simpler.

## 5 Conclusion

In conclusion, our new foraging module for the BEE‐STEWARD model facilitates the parameterization for new landscapes and the application of the model in general. This simplification is an important step toward a generic tool applicable to large spatial scales and a wide range of stakeholders. Thus, it can become a tool to guide practical land management and policy decisions, enhance research, and contribute to mitigating the ongoing decline of pollinators.

## Author Contributions

**Max Luttermann:** conceptualization (equal), formal analysis (lead), investigation (lead), methodology (equal), software (lead), writing – original draft (lead), writing – review and editing (equal). **Reinhard Prestele:** conceptualization (equal), supervision (supporting), writing – review and editing (equal). **Volker Grimm:** conceptualization (equal), supervision (supporting), writing – review and editing (equal). **Jürgen Groeneveld:** conceptualization (equal), formal analysis (supporting), investigation (lead), supervision (lead), writing – review and editing (equal).

## Conflicts of Interest

The authors declare no conflicts of interest.

## Data Availability

A Research Compendium, including the updated BEE‐STEWARD code with the new foraging module, as well as all data and analysis scripts, used to conduct the presented study can be found at https://doi.org/10.5281/zenodo.15401929.
